# Alexithymia impairs quality of life in irritable bowel syndrome

**DOI:** 10.2144/fsoa-2023-0068

**Published:** 2023-08-08

**Authors:** Mona Boudabbous, Ala Ben Issa, Ines Feki, Héla Gdoura, Lassad Chtourou, Manel Moalla, Rim Sallemi, leila Mnif, Ali Amouri, Jaweher Masmoudi, Nabil Tahri

**Affiliations:** 1 Gastroenterology Department, Hédi Chaker Hospital, Sfax, TUNISIA; 2 Phsychiatric Department, Hédi Chaker Hospital, Sfax, TUNISIA; 3 Sfax university of medicine, Tunisia

**Keywords:** alexithymia, irritable bowel syndrome, quality of life, SF-36, TAS 20

## Abstract

**Aim:**

Our objectives were to compare the frequency of alexithymia and the alteration of quality of life in irritable bowel syndrome (IBS) and to determine the factors associated with alexithymia and quality of life deterioration.

**Method:**

This is a comparative study which collected 80 IBS patients and 80 controls.

**Results:**

Quality of life was impaired in 75% of patients vs 37.5% (p < 0.0001). The prevalence of alexithymia was 50% in patients vs 1.2% (p < 0.0001). In multivariate analysis, an impaired quality of life was associated with alexithymia (p = 0.003). The factors associated with impaired quality of life were anxiety and alexithymia.

**Conclusion:**

Alexithymia was present in half of patients with IBS and its was associated with impaired quality of life.

Despite the continuous improvement of biomedical explorations and the multiplicity of research lines, the etiopathogeny of irritable bowel syndrome still remains poorly understood. Nevertheless, several physiopathological mechanisms are incriminated in its genesis. Among them, we find the impact of psychological factors and their repercussions on the quality of life of patients. In fact, IBS symptoms are sometimes exacerbated by stress and can be associated with psychological comorbidities. In patients with IBS, the most common comorbid psychiatric disorders seen include anxiety disorders (panic and generalized anxiety disorder), depression (including dysthymia), somatoform disorders (hypochondriasis and somatization disorder) and phobic disorders. Even if the prevalence of psychiatric disorders during the course of IBS is debatable, and it is sometimes difficult to distinguish between the primitive or secondary nature of mood disorders, linked to the impact of symptomatology on the quality of daily life. Psychological terrain and mood disorders are the primary conditions for access to care, and interfere with the perception and integration of sensory information of digestive origin, in particular by maintaining a state of hyper-vigilance to stimuli. In relation to a controlled population, there is a phenomenon of anticipation of pain during SII, which leads sufferers to experience pain for less intense stimuli. Bi-directional communication between the digestive tract and the central nervous system involves the pathways of the autonomic nervous system. Mediators of this autonomic system, notably catecholamines released by activation of the sympathetic system, modulate gastrointestinal function, affecting visceral sensitivity and intestinal immunity. Alterations in the functioning of this autonomic nervous system have already been reported during IBS. Psychological or mood disorders, frequent during IBS, may be responsible for disturbances in the activity of this autonomic nervous system. However, the association of psychoaffective disorders, including alexithymia, with IBS remains controversial in the literature. The evaluation of the importance of this association constitutes, however, an aid to the clinician both in understanding the pathogenesis of this disease and in improving its management.

Quality of life is defined by the WHO as “*An individual's perception of his or her place in existence, in the context of the culture and value system in which he or she lives, in relation to his or her goals, expectations, norms and concerns. It is a broad conceptual field, encompassing in a complex way the person's physical health, psychological state, level of independence, social relationships, personal beliefs and relationship with the specifics of their environment*” [1]. Because of its impact on quality of life, IBS is the cause of numerous psychosocial comorbidities which contribute to impairing the patient's daily life [2]. Alexithymia is characterized by an impaired ability to be aware of, explicitly identify and describe one's feelings. There is a decoupling of implicit and explicit emotional responses in alexithymia. Alexithymia was first described in patients seeking treatment for psychosomatic symptoms. This reduced emotional awareness could be expected to protect people with alexithymia from negative feelings, and possibly reduce anxiety and depression. However, the limited differentiation of emotional states in alexithymia actually seems to cause patients great difficulty in the regulation and resolution of negative affects. Consequently, the prevalence of affective disorders is greater in this population. Alexythymia has been associated with impaired quality of life in several pathologies, particularly psychosomatic ones such as fibromyalgia and migraine [3,4]. It even represents a real non-motor symptom in Parkinson's disease, causing a further deterioration in patients' quality of life [5]. In IBS, alexithymia was found to be a stable trait and a strong predictor of treatment outcome but its impact on quality of life was not investigated [6].

## Methods

This is a cross-sectional comparative case-control study. Our study population was divided into two groups: group 1 (G1): patients aged between 18 and 75 years with IBS collected at the gastroenterology outpatient clinic and having consulted between 1 October 2020 and 30 December 2020 and group 2 (G2): age- and sex-matched controls to patients. The diagnosis of IBS was based on the ROME IV criteria [7]. Informed consent was obtained from patients and controls.

The criteria for non-inclusion of patients were:

- Personal history of chronic gastrointestinal pathology other than IBS.
- Personal history of psychiatric pathology.
- Any serious chronic pathology (such as cancer) or disability: cardiological, respiratory, orthopedic, rheumatological and endocrinological.
- Presence of major cognitive impairment or mental retardation.
- For controls, in addition to the above criteria, controls should not have a history or symptoms of IBS.

All patients who consulted during the study period were included. Age- and sex-matched controls with no non-inclusion criteria were included. They were most often visitors or family members of the investigators or staff.

During the study period, we used an information sheet assessing sociodemographic, clinical, quality of life (SF-36), alexithymia (TAS 20) and anxiety disorders (HAD: Hospital anxiety and depression scale) scales.

The questionnaires were completed by a single operator who explained the items to the subjects included whenever necessary

### Sampling

The minimum population size was determined by the Schwarz formula (3): n = (i2 x p x q)/α2

i = 95% confidence level (i = 0.95);

p = estimated 24.5% prevalence of IBS from a previous study (4) (p = 0.245);

q = 1-p (q = 0.755);

α = margin of error at 5% (α = 0.05).

n = (0.952 × 0.245 × 0.755)/0.052;

n = (0,9025 × 0,245 × 0,755)/0,0025 = 0,1669399375/0,0025 = 67.

We added 10% to the number obtained by taking into account the response errors and non-responses to have the final number N of at least 74 cases and 74 controls.

### Statistical study

The computerized data entry and statistical analysis of the data were carried out with the statistical software Statistical Package for Social Sciences (SPSS) for Windows version 21. We expressed the qualitative variables in frequencies and the quantitative variables in means ± standard deviation (SD) after checking the normality of the distribution, or in median and interquartile range if the normality of the distribution was not checked. The Kolmogorov–Smirnov test was used to check the normality of the distribution of quantitative variables with a headcount ≥50. Comparison of percentages was done by the Chi-squared test when the conditions of application were verified, and by Fisher's test otherwise.

When the response variable was a two-modality qualitative variable, the comparison of means on unpaired series was done by the Student's test when the normality of the distribution was verified, and by the nonparametric Mann–Whitney–Wilcoxon test for unpaired series when this was not verified. When the response variable was an ordinal qualitative variable with more than two modalities, the nonparametric Kruskal–Wallis test was used. In all statistical tests, the significance level was set at 0.05 (p < 0.05). The multivariate analysis was based on binary logistic regression with adoption of the best models allowing a higher percentage of variables well classified in the univariate analysis with a statistical significance level of 0.1.

## Results

The mean age of the patients was 49.4 ± 10.6 years with extreme ages ranging from 30 to 69 years. There was no statistically significant difference in sociodemographic data between patients and controls (Table 1). The prevalence of alexithymia was 50% in patients vs 1.2% in controls with a statistically significant difference (p < 0.0001). The mean value of the alexithymia scale in patients was 58.14 ± 7 points vs 39.06 ± 12 points in controls with a statistically significant difference (p < 0.0001). Neither sociodemographic characteristics nor clinical features had influenced the occurrence of alexithymia.

**Table 1.** Comparison between patients and controls according to sociodemographic data.

	Patients	Controls	p-value
Sex feminine (%)	63.7	61,3	0.87
Mean age (years)	49.4 ± 10.6	47.02 ± 12.23	0.184
School level: n (%) Illiterate Primary Secondary University	11(13.8) 36 (45) 25 (31.3) 8 (10)	4 (5) 43 (53.8) 19 (23.8) 14 (17.5)	0.096
Professional status: n (%) No occupation Daily Middle management Retirement	35 (43.8) 25 (31.3) 11 (13.8) 9 (11.3)	31 (38.8) 20 (25) 14 (17.5) 8 (10)	0.084
Marital status: n (%) Bachelor Married Divorce Widower	10 (12.5) 67 (83.8) 0 3 (3.8)	10 (12.5) 68 (85) 1 (1.3) 1 (1.3)	0.571
Geographical origin: n (%) Urban Rural	48 (60) 32 (40)	58 (72.5) 22 (27.5)	0.132

The prevalence of impaired quality of life was high in the patients compared with the control group (75 vs 37.5%) with a statistically significant difference (p < 0.0001). The mean value of the SF-36 scale was 53.1 ± 18.4 points in patients versus 71.6 ± 11.7 points in controls with a statistically significant difference (p = 0.001). Comparison of the 2 groups (controls vs patients) according to the means of each quality of life domain showed that all domains were affected during IBS (Table 2). Low educational level and lack of work activity were associated with impaired quality of life in IBS patients, with a statistically significant relationship (p = 0.025). Other socio-demographic characteristics did not show a statistically significant influence on patients' quality of life. The presence of a family history of psychiatric illness, more severe abdominal pain and associated extra-digestive signs were factors associated with impaired quality of life with a statistically significant relationship (p = 0.044, p = 0.022 and p = 0.001, respectively) (Table 3). In addition, definite anxiety, major depressive disorder and alexithymia were associated with impaired quality of life in our patients (respectively: p = 0.001, p = 0.005 and p = 0.03). In multivariate analysis, impaired quality of life was associated only with the presence of alexithymia (p = 0.098) and definite anxiety (p = 0.024).

**Table 2.** Comparison between patients and controls according to quality of life scores.

Quality of life fields	Témoins	Patients	p-value
Physical function	74.1 ± 18.7	64.9 ± 23.5	<0.001
Limitations due to physical condition	64.4 ± 29.2	35.8 ± 31.2	<0.001
Physical pain	70.4 ± 27.6	36.7 ± 36.2	<0.001
Mental health	68.1 ± 8.7	57.3 ± 18.9	<0.001
Limitations due to mental state	72.8 ± 11.5	59.7 ± 17.2	0.037
Life and relationships with others	79 ± 14.6	65.8 ± 28.3	<0.001
Vitality	76.9 ± 13.9	49.6 ± 32	<0.001
Perceived health	66.6 ± 10.3	53.2 ± 16	<0.001
Total quality of life score	71.6 ± 11.7	53.1 ± 18.4	<0.001

**Table 3.** Alteration in the quality of life of patients according to their sociodemographic and clinical features.

Sociodemographic characteristics	Quality of life not altered	Quality of life altered	p-value
Mean age (years)	48.6 ± 8.4	51.2 ± 11.9	0.40
Sex Male Female	8 (10%) 9 (11.3%)	21 (26.3) 42 (52.5)	0.29
Geographical origin: n (%) Urban Rural	11 (13.8) 6 (7.5)	36 (45) 27 (33.8)	0.57
School level Low (illiterate or primary) High (secondary or university)	6 (7.5) 11 (13.8)	42 (52.5) 21 (26.3)	**0.019**
Professional activity: n (%) No Yes	6 (7.5) 11 (13.8)	40 (50) 23 (28.7)	**0.037**
Marital status: n (%) Do not live in couple Lives in couple	2 (2.5) 15 (18.8)	11 (13.8%) 52 (65%)	0.443
Mean age at onset of IBS (years)	36.4 ± 9.4	35.3 ± 12.7	0.55
Personal history of allergy	3 (3.8)	17 (21.3)	0.54
Personal history of asthma	1 (1.3)	9 (11.3)	0.68
Family history of IBS	13 (16.3)	40 (50)	0.31
Family history of psychiatric illnesses	0 (0)	**12 (15%)**	**0.044**
Smoking	2 (2.5)	16 (20%)	0.333
Alcohol consumption Occasional Chronic	1 (1.3%) 0 (0%)	4 (5%) 2 (2.5%)	0.766
Diarrhea, n (%) Constipation, n (%) IBS type Mixed (n%) Indeterminate, n (%)	0 (0) 12 (15) 2 (2.5) 3 (3.8)	5 (6.3) 42 (52.5) 13 (16.3) 3 (3.8)	0.190
Pain scale (EVA/10) Mean ± standard deviation	5.8 ± 1.1	**6.6 ± 1.3**	**0.022**
Accesses per month (n)	3.2 ± 2.4	4.4 ± 3.1	0.089
Location of abdominal pain, n (%) Periumbilical Colonic flanks and frame whole abdomen	8 (10) 8 (10) 1 (1.3)	20 (25) 20 (25) 15 (18.8)	0.330
Radiation of pain	8 (10%)	44 (55%)	0.081
Heartburn associated	3 (3.8%)	11 (13.8%)	0.617
Associated extra-digestive signs	10 (12.5%)	**59 (73.8%)**	**0.001**

Values in bold indicate statistically significant results.

IBS: Irritable bowel syndrome.

## Discussion

Alexithymia is characterized by difficulty identifying and describing feelings with an outwardly oriented thought pattern and limited imaginative capacity [8]. In addition to psychiatric illnesses (such as panic disorder and post-traumatic stress disorder), alexithymia has been associated with several pathologies, including cardiovascular disease, obesity, chronic pain, renal failure, eating disorders, and gastrointestinal disease [9].

A recent review of the literature examined the relationship between alexithymia and digestive disorders such as functional gastrointestinal disorders. In this review, 48 studies met the inclusion criteria. The prevalence of alexithymia was over two-thirds in functional digestive disorders, with a higher prevalence than in other digestive and liver diseases [10].

The prevalence of alexithymia was 50% in our patients vs 1.2% in controls. This result is close to an Indian study where the respective prevalences of alexithymia in patients and controls were 42% and 6.6% [11]. However, Jones *et al.* did not find a significant difference between patients and controls regarding the prevalence of alexithymia [12]. In our study, the mean value of the alexithymia scale in patients was 58.14 ± 7 points versus 39.06 ± 12 points in controls (p < 0.0001). This result is close to the values found in the literature [13–15]. Contrary to our results, alexithymia was correlated with the severity of IBS symptoms [13–15]. Alexithymia has been shown to have an impact on the response to treatment of IBS after control of gastrointestinal symptoms [6–16]. Alexithymia has been shown to be a stable factor and a stronger predictor of poor outcome of IBS treatment [17]. This fact would explain the negative impact of alexithymia on patients' quality of life found in our study. Due to the cross-sectional nature of our work, we were not able to study the impact of alexithymia on the response to treatment.

As found in our study, several authors have shown that IBS is associated with poor quality of life and impaired social functions [1,18,19]. The mean value of the SF-36 scale was 53.1 ± 18.4 points for our patients vs 71.6 ± 11.7 points for the controls with a statistically significant difference (p = 0.001). Our results are close to the results of the study by Addante *et al.* concerning the mean value of the SF-36 scale which was higher in 417 controls vs 290 patients (55.6 vs 44.8; p < 0.001) [20]. Similarly, in the study of Kanuri *et al.* the quality of life was significantly lower in the IBS population (n = 272) compared with the control population (n = 246; 50.4 ± 1.4 vs 63.8 ± 1.6; p < 0.001) [17].

Comparison of the two groups in our study (controls vs patients) according to the means of each quality of life domain showed that all domains were affected during IBS. This same result was also obtained in the study of Mahassadi *et al.* where IBS patients had low SF-36 scores in all eight domains compared with control subjects [21].

In our study, a low level of education and the absence of professional activity were associated with an altered quality of life in patients with IBS, with a statistically significant relationship (p = 0.025). These precarious living conditions are probably at least partly responsible for affective disorders that impair quality of life. In the social sciences, macrosystems is defined as the representation of major cultural trends, while microsystems reflect these trends but are linked to smaller units such as the individual, the family, the school or rehabilitation programs. These microsystems reflect the culture in general, and embody the values, laws and paradigms, or ways of thinking and organizing information, of that culture. The concept of quality of life is an interesting one, since it embodies both the notion of a ‘macrosystem’ (‘my country's quality of life’) and the notion of a ‘microsystem’ (‘my own quality of life’). Quality of life is based on a set of values that value the strengths of the individual and his or her family. Quality of life is determined by congruent social values and behaviors. Objective factors measuring environmental conditions such as health, well-being, friendship, standard of living, education, public safety, housing, neighbourhood and leisure are indices that enable us to make clear, concise and balanced judgements about conditions relating to important aspects of society. In this context, the level of education and professional activity could influence patients' quality of life [22].

Moreover, definite anxiety and alexithymia were independent factors associated with impaired quality of life in our study. These data are consistent with the results of another study where the most important predictors of poor quality of life were anxiety and depressive symptoms related, according to these authors, to perceived gastrointestinal symptoms in IBS (p < 0.01) [20]. In addition, the presence of a family history of psychiatric illnesses, more intense abdominal pain and associated extra-digestive signs were factors associated with impaired quality of life in our patients with a statistically significant relationship. Consistent with our findings, in the study by Coffin *et al.*, there was a significant correlation (p < 0.001) between the intensity of IBS symptoms and impaired quality of life. However, the other factors significantly correlated with quality of life impairment, which were the type of IBS and gender, were not found in our study [23]. In another study of 149 IBS patients, the authors noted that the SF-12 physical and SF-12 mental components were negatively associated with the intensity of IBS symptoms. The SF-12 physical components were also negatively associated with the number of organic comorbidities and a shorter school career, and the SF-12 mental components were negatively associated with neuroticism and smoking. According to these authors, somatization and, to a lesser extent, organic comorbidities were independent predictors of reduced physical quality of life, and affective disorders and neuroticism predicted reduced mental quality of life. They noted that the cause of reduced physical and mental quality of life differed and that the IBS symptom intensity score predicted neither physical nor mental quality of life after adjustment for comorbidity [24].

## Limitations

This study is subject to the limitations of its observational design; in particular, we were unable to establish causal relationships between IBS symptom severity and quality of life.

Furthermore, we did not discuss the efficacy of the treatment used in our population. Furthermore, we believe that other studies evaluating the effect of medication on improving quality of life and reducing alexythmias would be useful.

The study populations (patients and controls) were selected from a hospital community, in a tertiary center, which may limit the generalizability of our results.

However, our results are useful for highlighting potential therapeutic targets, such as psychoaffective disorders, which, when managed, patients will regain a quality of life comparable to that of controls without IBS.

## Conclusion

Despite its association with several organic pathologies and its presence in 50% of our patients with IBS, alexithymia is a disorder little known by clinicians and therefore rarely investigated in practice. However, this disorder is associated with anxiety and contributes to the impairment of quality of life. We recommend the systematic search for alexithymia and the assessment of quality of life in these patients, especially those with identified social factors and severe symptoms.

Earlier identification of at-risk patients will facilitate targeted treatments of specific factors of poor quality of life in IBS. To adequately assess and manage the impact of IBS, timely assessment of quality of life and initiation of treatment when it is impaired should be a priority. Thus, individualized treatment based on the type and cause of quality of life impairment should be targeted to IBS patients to improve their quality of life, not just relieve their symptoms.

**Summary points**

- Compared with controls, patients with irritable bowel syndrome had more impaired quality of life, higher mean values of the alexithymia scales, and higher frequency of alexithymia.
- Low educational level and lack of work activity were associated with impaired quality of life in irritable bowel syndrome (IBS) patients, with a statistically significant relationship (p = 0.025).
- Neither sociodemographic characteristics nor clinical features had influenced the occurrence of alexithymia.
- In multivariate analysis, impaired quality of life was associated only with the presence of alexithymia (p = 0.098) and definite anxiety (p = 0.024).
- We recommend that gastroenterologists and other physicians, who are responsible for the treatment of IBS patients, also pay attention to and evaluate the concomitant presence of alexithymia and consider specialized treatment for this disorder.

## References

[1] World Health Organization. Quality of Life Assessment: An Annotated Bibliography. WHO, Geneva, Switzerland, 32 (1994). (WHO/MNH/PSF/94.1). https://apps.who.int/iris/handle/10665/61629?locale-attribute=en&WHO_MNH_PSF_94.1.pdf

[2] Brun-Strang C, Dapoigny M, Lafuma A, Wainsten JP, Fagnani F. Irritable bowel syndrome in France: quality of life, medical management, and costs: the Encoli study. Eur. J. Gastroenterol. Hepatol. 19(12), 1097–1103 (2007). 17998835 10.1097/MEG.0b013e3282f1621b

[3] Tesio V, Di Tella M, Ghiggia A Alexithymia and Depression Affect Quality of Life in Patients With Chronic Pain: A Study on 205 Patients With Fibromyalgia. Front. Psychol. 4, 9–442 (2018). 10.3389/fpsyg.2018.00442 PMC5893813 29670558

[4] Vieira RVdA, Vieira DC, Gomes WB Alexithymia and its impact on quality of life in a group of Brazilian women with migraine without aura. J. Headache Pain. 14, 18 (2013). 23565860 10.1186/1129-2377-14-18 PMC3620425

[5] Klietz M, Schnur T, Drexel SC Alexithymia Is associated with reduced quality of life and increased caregiver burden in Parkinson's disease. Brain Sci. 10(6), 401 (2020). 32599704 10.3390/brainsci10060401 PMC7348697

[6] Porcelli P, De Carne M, Leandro G. The role of alexithymia and gastrointestinal-specific anxiety as predictors of treatment outcome in irritable bowel syndrome. Compr. Psychiatry 73, 127–135 (2017). 27940317 10.1016/j.comppsych.2016.11.010

[7] Mearin F, Lacy BE, Chang L Bowel disorders. Gastroenterology 150(6), 1393–1407 (2016). 10.1053/j.gastro.2016.02.031 27144627

[8] Nemiah JC. Alexithymia: une vision du processus psychosomatique. Mod. Trends Psychosom. Med. 3, 430–439 (1976).

[9] Taylor GJ, Bagby RM, Parker JD. Disorders of affect regulation: Alexithymia in medical and psychiatric illness. Cambridge University Press (1999). 10.1017/CBO9780511526831

[10] Carrozzino D, Porcelli P. Alexithymia in Gastroenterology and Hepatology: A Systematic Review. Front Psychol. 9, 470 (2018). 29681874 10.3389/fpsyg.2018.00470 PMC5897673

[11] Arun P. Alexithymia in irritable bowel syndrome. Indian J. Psychiatry. 40(1), 79–83 (1998). 21494449 PMC2964824

[12] Jones MP, Wessinger S, Crowell MD. Coping strategies and interpersonal support in patients with irritable bowel syndrome and inflammatory bowel disease. Clin. Gastroenterol. Hepatol. Off. Clin. Pract. J. Am. Gastroenterol. Assoc. 4(4), 474–481 (2006). 10.1016/j.cgh.2005.12.012 16616353

[13] Huang JS, Terrones L, Simmons AN, Kaye W, Strigo I. A Pilot Study of fMRI Responses to Somatic Pain Stimuli in Youth with Functional and Inflammatory Gastrointestinal Disease. J. Pediatr. Gastroenterol. Nutr. 63(5), 500–507 (2016). 27574880 10.1097/MPG.0000000000001390 PMC5074879

[14] Phillips K, Wright BJ, Kent S. Psychosocial predictors of irritable bowel syndrome diagnosis and symptom severity. J. Psychosom. Res. 75(5), 467–474 (2013). 24182637 10.1016/j.jpsychores.2013.08.002

[15] Farnam A, Somi MH, Farhang S, Mahdavi N, Ali Besharat M. L'effet thérapeutique de l'ajout d'une formation à la conscience émotionnelle au traitement médical standard du syndrome du côlon irritable: un essai clinique randomisé. J. Psychiatr. Pract. 20(1), 3–11 (2014). 24419306 10.1097/01.pra.0000442934.38704.3a

[16] Portincasa P, Moschetta A, Baldassarre G, Altomare DF, Palasciano G. Pan- enteric dysmotility, impaired quality of life and alexithymia in a large group of patients meeting ROME II criteria for irritable bowel syndrome. World J. Gastroenterol. 9(10), 2293–2299 (2003). 14562396 10.3748/wjg.v9.i10.2293 PMC4656481

[17] Porcelli P, De Carne M, Leandro G. The role of alexithymia and gastrointestinal-specific anxiety as predictors of treatment outcome in irritable bowel syndrome. Compr. Psychiatry 73, 127–135 (2017). 27940317 10.1016/j.comppsych.2016.11.010

[18] Kanuri N, Cassell B, Bruce SE L'impact de l'abus et de l'humeur sur les symptômes intestinaux et la qualité de vie liée à la santé dans le syndrome du côlon irritable (SCI). Neurogastroenterol Motil. Off. J. Eur. Gastrointest. Motil. Soc. 28(10), 1508–1517 (2016). 10.1111/nmo.12848 PMC5042818 27151081

[19] Bouchoucha M, Hejnar M, Devroede G, Babba T, Bon C, Benamouzig R. Anxiété et dépression comme marqueurs de la multiplicité des sites des troubles gastro-intestinaux fonctionnels: une question de genre? Clin. Res. Hepatol. Gastroenterol. 37(4), 422–430 (2013). 23270854 10.1016/j.clinre.2012.10.011

[20] Addante R, Naliboff B, Shih W Predictors of health-related quality of life in irritable bowel syndrome patients compared with healthy individuals. J. Clin. Gastroenterol. 53(4), 142–149 (2019). 29351154 10.1097/MCG.0000000000000978 PMC6051929

[21] Mahassadi AK, Ebela PC, Bangoura AD, Attia AK. Le poids du syndrome du côlon irritable et de la constipation chronique sur la qualité de vie liée à la santé chez les Africains noirs: une comparaison avec des sujets témoins sains en Côte d'Ivoire, Afrique de l'Ouest. Clin. Exp. Gastroenterol. 12, 355–365 (2019). 31447575 10.2147/CEG.S192563 PMC6684486

[22] Schalock RL. La qualite de vie: conceptualisation, mesure et application. Revue francophone de la déficience intellectuelle. 4(2), I37–I5 (1993).

[23] Coffin B, Dapoigny M, Cloarec D, Comet D, Dyard F. Relation entre la sévérité des symptômes et la qualité de vie chez 858 patients atteints du syndrome du côlon irritable. Gastroenterol. Clin. Biol. 28(1), 11–15 (2004). 15041804 10.1016/s0399-8320(04)94834-8

[24] Michalsen VL, Vandvik PO, Farup PG. Prédicteurs de la qualité de vie liée à la santé chez les patients atteints du syndrome du côlon irritable. A cross-sectional study in Norway. Health Qual. Life Outcomes. 13, 113–122 (2015). 26223784 10.1186/s12955-015-0311-8 PMC4518561

